# Advances in the In Vivo Quantitative and Qualitative Imaging Characterization of Gliomas

**DOI:** 10.3390/cancers14143324

**Published:** 2022-07-08

**Authors:** Pierpaolo Alongi, Ignazio Gaspare Vetrano

**Affiliations:** 1 Nuclear Medicine Unit, A.R.N.A.S. Ospedale Civico Di Cristina Benfratelli, 90127 Palermo, Italy; 2 Department of Neurosurgery, Neuro-Oncological Surgery Unit, Fondazione IRCCS Istituto Neurologico Carlo Besta, 20133 Milan, Italy; ignazio.vetrano@istituto-besta.it

Gliomas are the most common and aggressive intra-axial primary tumours of the central nervous system (CNS), arising from glial cells. The poor prognosis of these tumours partly results from a lack of significant advances in early diagnosis and treatments prolonging life [1].

Most of the available local treatment options, including radiotherapy and neurosurgery, heavily depend on precise knowledge of the type, location, and extent of the tumour. Visual assessments of medical imaging represent a unique possibility for the detection, characterization, and monitoring of diseases. Qualitative evaluations still represent the modality for intraoperative assessments using ultrasound (ioUS) and/or optical coherence tomography (OCT) to identify lesions and potential residue, analyse the vascularization pattern, and characterise the nature of the mass. Technological advances in both structural and functional imaging (MR and PET imaging) also enables defining several images features, through multiparametric and quantitative evaluations of gliomas [2,3].

In the field of quantitative imaging, the main issues regard the acquisition/reconstruction parameters, tissue segmentation, feature extraction/selection, and the appropriate statistical analysis of data [4].

Radiomics analysis, considered to be the most advanced development inimage quantification, may provide valuable diagnostic, prognostic, and predictive information of tumours. However, its role in gliomas is still undefined. Numerous studies have demonstrated the correlation between the heterogeneity of the tissues and the radiomics features, which can obtain relevant information not conventionally assessable in medical imaging [5,6]. Future perspective studies will be necessary to discover new possible correlations among clinical, genetic/molecular, and image features of gliomas.

There is still a need to validate the advanced use of MR and PET techniques, as the diagnostic methods increasingly utilized in clinical and research settings [7,8,9,10]. Potential advantages are still to be confirmed on the evaluation of gliomas, to define the progression of disease and for monitoring high-grade gliomas [11,12].

Diagnoses, disease extensions, vascularization patterns, differential diagnoses between low-grade and high-grade gliomas, and definitions of disease status after treatment remains the main issues of medical imaging in gliomas. This Special Issue will focus on developing in vivo quantitative and qualitative imaging (CT, OCT, MRI, PET/CT, and PET/MR), also considering the novel application of radiomics-based analysis to improve the current role of imaging in the management of gliomas.

We invite authors to submit contributions of novel findings or reviews that comprehensively highlight the latest discoveries in the field.
